# Sex differences in high-level appreciation of automobile design-evoked gamma broadband synchronization

**DOI:** 10.1038/s41598-020-66515-7

**Published:** 2020-06-17

**Authors:** Regina W. Y. Wang, Tsai-Miau Ke, Shang-Wen Chuang, I-Ning Liu

**Affiliations:** 1 Design Perceptual Awareness Lab (D:pal), National Taiwan University of Science and Technology (Taiwan Tech), Taipei, Taiwan; 2 Department of Design, National Taiwan University of Science and Technology, Taipei, Taiwan; 3 Taiwan Building Technology Center, National Taiwan University of Science and Technology (Taiwan Tech), Taipei, Taiwan

**Keywords:** Decision, Biomedical engineering

## Abstract

The present study was conducted to provide neuroimaging correlates for neurodesign of automobile for marketing aesthetics, using event-related spectral perturbations (ERSPs) and participant reports. Thirty men and women aged 22–27 years were presented with various 3-dimensional automobile modelling shapes (rectangular, streamlined, and round), which were cross-matched with various interior colour tones (pure hue/vivid, light, and dark tones) in the experimental conditions, i.e., rectangular exterior with a vivid tone interior. The stimuli pairs were to be rated by participants to facilitate our understanding of the emotional dimensions of automotive design qualities. Significant differences were observed in the high gamma band of 80–100 Hz in the left temporal area between the two sexes. Men elicited a stronger high gamma band signals for dark colour tone interiors and rectangular or round automobile modelling designs because of the meaningful and comprehensible signals associated with the mechanisms of working memory. In contrast, women had fewer reactions than men, and elicited higher beta-band dynamics in the anterior cingulate cortex for rectangular automobile modelling design, and higher gamma-band dynamics for light colour tone interiors, which might relate to their higher self-awareness of positive emotional reward.

## Introduction

The sex of the person is an innate division in human physiological functions, which would be related to the division of labour and biological evolution of human life. It has been a key topic in different fields of neurobiology, psychology, personality and sociality. Innate factors include emotions, memory, perception, language, and other fields. Males utilize the spatial brain regions and mechanical tasks to learn, displaying a tendency toward visual or practical learning, whereas females are better at applying the language and emotional functions of the brain^1^. Research has shown that the early hunter role of males has enabled them to develop greater perception of spatial positioning^2^ and superior three-dimensional 3-D spatial awareness^3^. By contrast, the early gatherer role of females enabled them to have greater abilities to perceive visual features^4^, while the female role of nurturing children enabled them to have higher emotional sensitivity^5^. Acquired factors refer to the gender roles developed due to the influence of different countries, ethnicities, and other cultural and social roles. In traditional societies, male personalities are manifested as masculine, representing power and the responsibility to support their families, whereas females give the impression of being vulnerable and in need of protection. These concepts were formed owing to the development of gender roles learned by role-modelling parents and peers, which in turn led to the formation of gender stereotypes. Hence, humans display a gender division in expertise or interest from a young age. For example, boys should wear blue clothes and like playing with machines and cars, whereas girls should have pink things and like playing house and playing with dolls^6^. Several studies have also found that gender stereotypes led to sex differences in self-construal, such that most males showed higher independence in their self-construal, whereas females showed higher interdependence^7^. Males focused on the functional requirements during the purchasing process, with an emphasis on the content and technical information of products^8^; they mainly considered their needs when purchasing. By contrast, females focused on emotional and experiential satisfaction, with a greater emphasis on evaluating information, including identity-related issues, rather than merely product-related content^8^. They were more concerned about multiple factors with a focus on sensibility and emotional richness^8,9^.

Emotion research related to the gender of the person found that positive and scenery clips elicited higher levels of pleasantness in both males and females, whereas neutral or negative film clips elicited greater unpleasantness (compassion, sadness, and fear) in females^10^. A functional magnetic resonance imaging (fMRI) study on sex differences also showed that viewing disgust-inducing, fear-inducing, and neutral pictures evoked stronger brain responses in females than in males^11^. Aesthetic visuals can help to induce pleasant emotions in humans^12^. Visual stimulation with beautiful products (shape, proportions, colour, and material) and artworks elicited higher reward value and pleasure in the prefrontal cortex than that with ugly stimuli^13,14^. The *Oxford Dictionary* defined aesthetics as ‘a set of principles concerned with the nature of beauty’ (*Oxford Dictionaries* online)^15^. Judgments of design aesthetics rely on various neural mechanisms, including human cognitive, emotional and reward processing, and even embodied experience^16^. Carl Gustav Jung proposed that aesthetics is derived from the collective unconsciousness of humans^17^. The present study posits that the different aesthetic factors of product design in different sexes may lead to differences in sensation concern of event-related dynamics in the EEG spectrum. This can be a key exploration of young issue in neurodesign concern of affective and decision neuroscience and biomedical engineering.

Audi pointed out that up to 60% of a consumer’s decision to purchase cars is made on their appearance, rather than their internal technical performance^18^. Thus, the topic of aesthetics is prominent in the development of marketing strategies for automobile products^19–21^. The process of automotive design involves aesthetics, ergonomics, and environmental requirements as well as the automotive interiors^22^. It is performed by designers who generally have an art background in industrial design or transportation design. This study considered the perspectives of the modelling/drawing processes in automotive design, i.e., the application of design techniques in creating various automotive categories^23,24^. The demand during the mature, declining stages of the auto industry has led automotive producers to increase their product variety (introducing new versions) rather than to introduce product innovations (new engines, restyling, new models) due to standardization and modularization since 1984–2012^25,26^. The increased variety of paintwork, headlight, and wheel designs in the automobile exteriors or seat, trim, and interactive control panel designs in the interiors were found to be useful to maintain high standards of quality management^27^. A car research begins on the computer screen, particularly through the official websites of different automobile manufacturers. Meeting the consumers’ desires appeals to the use of digital contents displaying various models and designs, which enhances the readers’ or customers’ engagement, a process that is more crucial than ever for increasing profitability^28^.

**Figure 1:**
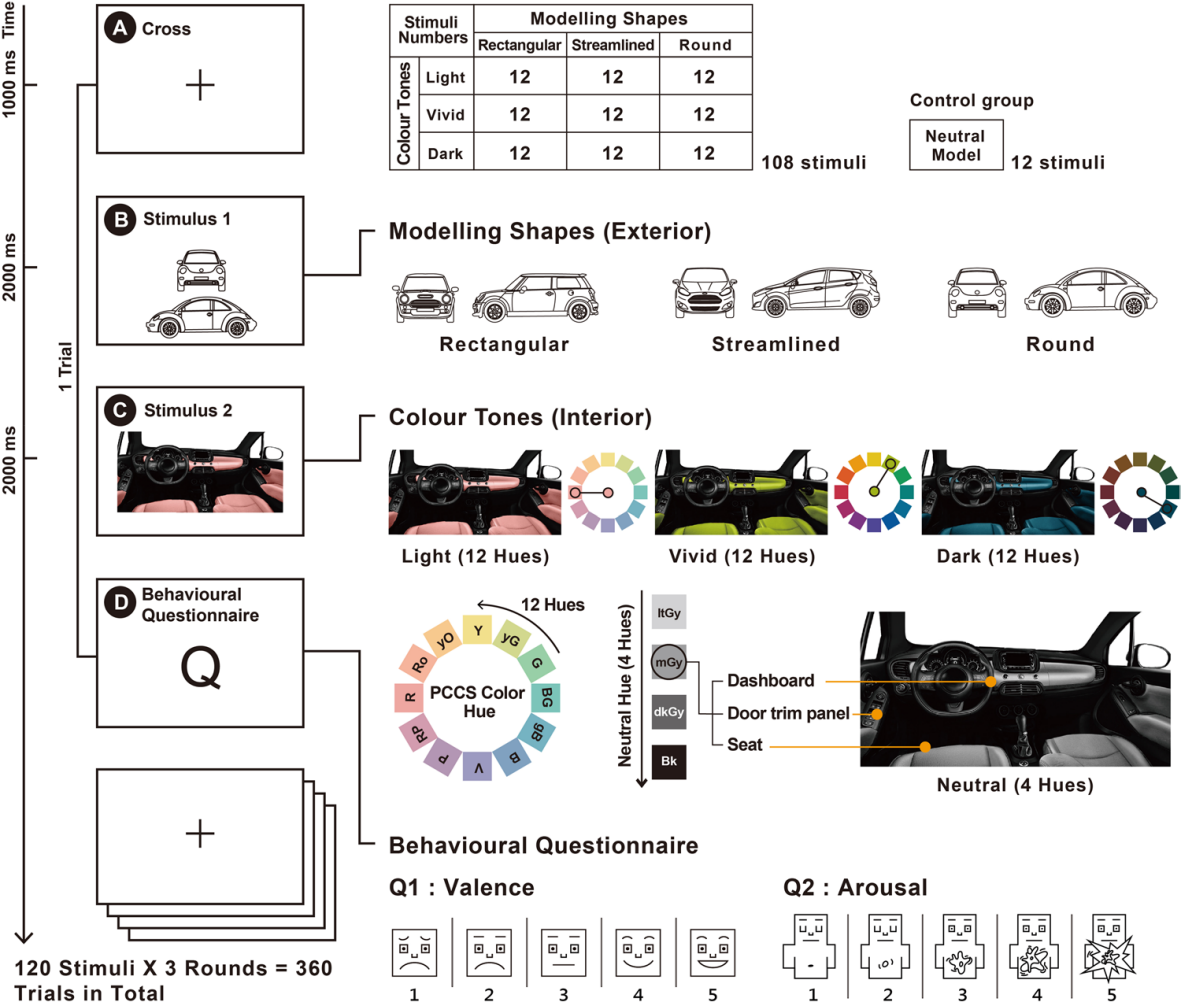
(**A**) This experiment involves random presentation of stimuli containing 3-dimensional shapes of automobile modelling combined with interior colour tones (1024 x 768 pixels). The experimental procedure involved the presentation of the following: First, a ‘+’ fixation point for 1000 ms; second, the shapes of the automobile modelling for 2000 ms; finally, the interior colour tones for 2000 ms. Thereafter, the behavioural questionnaire was presented; the trial was completed after the participant gave his responses to the questionnaire. The stimuli at the 10 conditions were presented to 9 experimental groups and 1 control group. Each condition had 12 stimuli. Therefore, 120 stimuli were repeated thrice to include a total of 360 trials and 36 trials in each condition (120 stimuli × 3 times = 360 trials/10 conditions = 36 trials per condition). The experiment lasted approximately 45 minutes. (**B**) The 3D shapes of the automobile modelling included (i) rectangular (little curved), represented by the MINI ONE; (ii) streamlined (medium curved), represented by the BMW 1 series; and (iii) round (extremely curved), represented by the Volkswagen Beetle. (**C**) Three colour tones were proposed as follows: (i) vivid tone (pure hue without the addition of grey), (ii) light tone (with the addition of light grey), and (iii) dark tone (with the addition of dark grey). Each tone used was included in the Practical Color Coordinate System (PCCS) 12 hues (R, Ro, yO, Y, yG, G, BG, gB, B, V, P, and RP) and formed the experimental group. The neutral colour tones included 4 hues (itGy, mGy, dkGy, and Bk), which formed the control group. (**D**) The behavioural questionnaire included emotional valence and emotional arousal. The options were designed by the Likert 5-point scale and the Self-Assessment Manikin (SAM). A score of ‘1’ represented the lowest level, and ‘5’ indicated the highest level.

This study suggested that the ‘3D shape of automobile modelling’ and ‘tone of interior colour’ constitute the nature of automobile aesthetics that are associated with consumers’ emotional reactions^29,30^. The shapes of the automobile models in this study referred to the design of the product’s external 3D shapes, including surface contour features such as straight lines, curves, and fillets^31^. Three vehicle modelling shapes were included in the experiment (Fig. 1B): ‘rectangular’ (few curves), ‘streamlined’ (moderately curved), and ‘round’ (highly curved). The features of ‘rectangular’ vehicle modelling were straight and small filleted corners and rectangular contours with little curved surfaces, which are often utilized in the RV, Sedan, Wagon, Luxury cars, MPV, or Van categories, represented by Discovery (2012), Mini One (2011), Land Rover, and Suzuki Landy (2010). The features of ‘streamlined’ vehicle modelling encompassed the curves of aero-flowing and aerodynamic designs, i.e., streamlined contours with moderately curved surfaces, often utilized in the sports car or supercar category, represented by BMW 1 Series (2019), Audi R8 (2013), and Peugeot 206 (2013). The features of ‘round’ vehicle modelling included rounded contours and filleting with large curved surfaces, often utilized in the compact or city car category, represented by the Mercedes-Benz Smart (2017) and VW Beetle (2013).

The 3D shapes design of automobile modelling not only involves exterior; colour is also another important attribute. Different psychological attributes are sensitive to the aesthetic perception of colours^32^. Research into the different sexes has shown that women were more likely to perceive colours than men^33^ and were more attracted to brighter colours^34,35^; women were also more sensitive to different colour shades and stylistic patterns^36,37^. The independent variable in this study was the colour tone of the Practical Color Coordinate System (PCCS), which was developed by the Japan Color Research Institute in 1964^38^. PCCS is a renowned colour order system, which includes attributes of the other three well known systems, Munsell, NCS (Natural Color System) and Ostwald. PCCS invented ‘tone’ as a new parameter of colour attribute, which summarizes saturation (chroma) and lightness (value). Each of the colour tones can present as 12 Hues (R, Ro, yO, Y, yG, G, BG, gB, B, V, P, and RP). In colour theory, tone is any pure hue with neutral grey (a mixture of white and black) added^39^. The colour hue, therefore, remains the same except it is less bright. The three interior colour tones used in the experiment (Fig. 1C) included ‘vivid tone’, a pure colour hue without neutral grey added; ‘light tone’, a pure colour hue with light neutral grey added); and ‘dark tone’, a pure colour hue with dark neutral grey added. Different from the other colour systems, the PCCS can effectively communicate the emotions of colour^40^. It provides an effective way to work with different colour matching and colour combinations, therefore, has an aesthetical and commercial value^41^. Different colour hues of the same tone complement each other better (i.e., a light blue tone and a light camel tone work better than dark blue and light camel tones). Three tones of colour, such as light, vivid, and dark tones, were proposed as independent variables in this study, which included high, moderate, and low levels of values and chroma.

The dependent variables of this study were emotional valence and emotional arousal. Emotions are generated on the basis of subjective perceptions and objective responses under external stimulation^42^. Past studies suggested that aesthetic preference depends on reward processing emotional evaluation. The physiological responses, behavioural manifestations, and cognitive behaviours elicited by emotions are regarded as continuous states of evocation, activation, and motivation. These have a key influence on human comprehension of events and objects, and plays a dominant and crucial role. The psychologist Russell measured emotions by using two dimensions, emotional valence and emotional arousal. Emotional valence refers to the positive and negative aspects of emotions, that is, the level of ‘pleasantness-unpleasantness’. Emotional arousal refers to the intensity of emotions, that is, the level of ‘excitement-calmness’^43,44^. Past studies that used the International Affective Picture System (IAPS) and Ekman’s Pictures of Facial Affect (POFA) found that positive and negative emotional stimuli evoked greater brain activation than neutral stimuli^45,46^. Positive emotional stimuli can bring about pleasant feelings in humans (cute animals, babies, etc.), and negative emotional stimuli can induce unpleasant feelings (scenes of attack, devastation, etc.), whereas neutral emotional stimuli will elicit relaxed and normal feelings (house, people, etc.)^47^. The subjective feelings of aesthetics are based on the emotional judgments perceived by the individual (i.e., emotions and self-related explanations), while the objective responses of aesthetics are achieved through the analysis of relevant information (emotional analysis, clear classification, cognitive processing, etc.).

With the progress of neuroscience, electroencephalography (EEG) experiments can record the brainwave activities of participants when continuously viewing stimuli. This type of noninvasive electrophysiological monitoring technology has also been applied to understand consumers’ responses to marketing stimuli^39,40^. Studies on gender aesthetics have shown significant differences in the parietal region when different sexes were judging the same beautiful artwork or natural visual stimuli^2^. A study on the allocation of visuospatial attention (left and right arrows) found that the left-hemispheric temporal and parietal regions of males showed enhanced activation in task selection, whereas the right-hemispheric inferior frontal gyrus and temporal regions of females showed greater activation^48^. Many studies on neuroaesthetics have found that an aesthetic judgement task involving decision-making is based on reward and emotion networks. Most of them involve regions of the brain, namely the orbitofrontal cortex (OFC), anterior insula, and anterior cingulate cortex (ACC)^16,49,50^. The OFC is noted for its role in reward processing, in terms of appraisal of an object’s qualities, whereas the anterior insula processes responses from the introspective cortex. ACC is associated with the appraisal of the outcomes and emotional monitoring underlying the effective connectivity of the OFC and anterior insula. This suggests that emotional salience monitoring is the prediction and intention of aesthetic processing. Aside from evoking the frontal lobe, visual features, such as target identification of shape and colour, can also activate the early visual areas in the occipital region^51,52^. EEG results of the letter identification task indicate that males exhibit greater theta- and alpha-band activities than females in the right temporoparietal region^53^. Comprehension involves triggering the visual areas, and beta and gamma responses in the semantic area. Meaningful stimuli will evoke a stronger gamma band^54,55^.

This study aimed to provide new information on the functions of the brain and neurodesign in automobile marketing. This will help in the design of elements that appeal to human beings in general, and to understand consumer behaviour and decision-making by gaining insight into the subconscious responses to design. This is in contrast to traditional methods, which help determine the optimum design elements for a specific participant through self-reporting questionnaires^56,57^. Certain large-scale companies, e.g., Coca-Cola, Tesco, and Google, use neuroscience research techniques to improve their product contents^28,58^. Optimizing design content for marketing will always involve a variety of methods. This study focused on young adult men and women of the ages of 22–27, in combination with a luxury brand of car, such as Mercedes, which has planted seeds of desire very early in a consumer’s life by creating specific campaigns, particularly online^59^. To facilitate validation of the data and hypotheses of the present study through cross-verification from different sources, this study on neurodesign of automobile marketing and aesthetics used multiple methods, including psychophysiological measurements of brain activity, participant self-report, and a literature review. Moreover, correlations between self-reported data and psycho-physiological data have been observed in a variety of domains, such as mobile interaction, simulation, human-robot interaction, and games^60^. The experimental design, stimuli, and procedures are shown in Fig. 1. The first hypothesis (H1) of the study was as follows: different 3D automobile modelling shapes, ranging from rectangular to rounded, evoke higher event-related dynamics in the EEG spectrum with respect to sex differences in emotion. The second hypothesis (H2) of the study was as follows: different interior colour tones, ranging from pure hue without added grey to pure hue with added dark grey, evokes higher event-related dynamics in the EEG spectrum with respect to sex differences in emotion.

## Results

### Behavioural results

**Figure 2:**
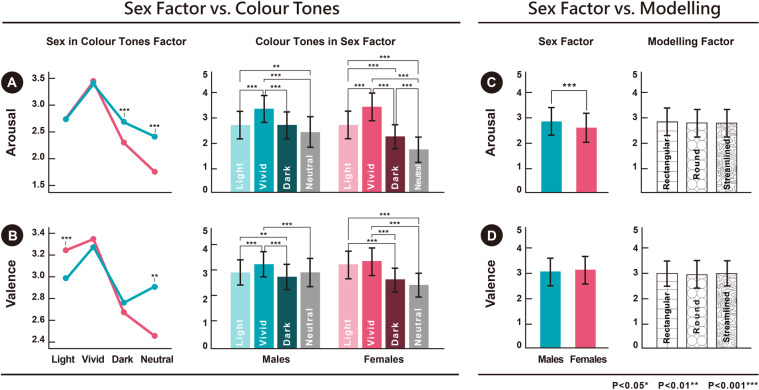
The behavioural data of the 30 participants toward the stimuli showed the following: (**A**) Males had higher emotional arousal than females for dark and neutral colour tones (p < 0.001). The emotional arousal of males toward interior colour tones were as follows: light > neutral; vivid > light, vivid > dark or vivid > neutral. The emotional arousal of females toward interior colour tones were as follows: light > dark, light > neutral; vivid > light, vivid > dark, vivid > neutral, or dark > neutral. (**B**) Females had higher emotional valence than males for light colour tones (p < 0.001); males had significantly higher emotional valence than females for neutral colour tones (p < 0.01). The emotional valence of males toward interior colour tones were as follows: light > dark; vivid > light, vivid > dark or vivid > neutral. The emotional valence of females toward interior colour tones were as follows: light > dark, light > neutral; vivid > dark or vivid > neutral. (**C**) The emotional arousal of males was significantly higher than that of females for all types of automobile modelling (p < 0.001). (**D**) The emotional valence of males and females were not significantly different for all types of automobile modelling (p > 0.05).

Sex differences and ‘interior colour tone’ had an interaction effect on ‘emotional arousal’ [F (3, 3591) = 18.128, p = 0.000, η²p = 0.015]. Hence, a simple main-effect test and Bonferroni post hoc comparison were performed. Under dark colour tones, the emotional arousal of males (M = 2.72, SD = 1.054) was higher than that of females (M = 2.31, SD = 0.918), [F (1, 1077) = 46.105, p = 0.000]; under neutral colour tones, the emotional arousal of males (M = 2.48, SD = 1.396) was higher than that of females (M = 1.73, SD = 1.091), [F(1, 358) = 32.240, p = 0.000]. The emotional arousal of both males and females was significantly higher for vivid colour tones than for other colour tones (dark, and neutral; Fig. 2A). Sex differences and ‘interior colour tone’ had an interaction effect on ‘emotional valence’ [F (3, 3591) = 15.171, p = 0.000, η²p = 0.013]. Hence, a simple main-effect test and Bonferroni post hoc comparison were performed. Under light colour tones, the emotional valence of females (M = 3.22, SD = 1.110) was higher than that of males (M = 2.92, SD = 1.009), [F (1, 1078) = 21.870, p = 0.000]; under neutral colour tones, the emotional valence of males (M = 2.91, SD = 1.207) was higher than that of females (M = 2.42, SD = 0.974), [F (1, 358) = 17.889, p = 0.000]. The emotional valence of both males and females was significantly higher for vivid colour tones than for other colour tones (eg., light or neutral, Fig. 2B).

Sex differences and ‘automobile modelling’ had no interaction effect on ‘emotional arousal’ [F (2, 3593) = 0.803, p = 0.448, η²p = 0.000]. Hence, a main-effect test and Bonferroni post hoc comparison were performed on the two factors, ‘sex’ and ‘automobile modelling’; sex differences had a significant main-effect; males (M = 2.91, SD = 1.154) had higher emotional arousal than females (M = 2.72, SD = 1.199), [F (1, 3593) = 22.668, p = 0.000]. The different types of automobile modelling did not have a significant main-effect on ‘emotional arousal’ [F (2, 3593) = 0.105, p = 0.901] (Fig. 2C). Sex differences and ‘automobile modelling’ had no interaction effect on ‘emotional valence’ [F (2, 3593) = 0.598, p = 0.550, η²p = 0.000]. Hence, a main-effect test and Bonferroni post hoc comparison were performed on the two factors, ‘sex’ and ‘automobile modelling’. The different sexes [F (1, 3593) = 1.732, p = 0.188] and automobile modelling [F (2, 3593) = 0.213, p = 0.808] did not have significant differences on ‘emotional valence’ (Fig. 2D).

The first hypothesis (H1), 3D shapes of automobile modelling evoke higher event-related dynamics in the EEG spectrum with respect to the sex differences in emotion, was partially verified by the behavioural results in Fig. 2C. The main effects demonstrate that the emotional arousal of males was significantly different and higher than females for the 3D shapes of automobile modelling overall. However, each model was not distinctive from the others. The results of the behaviour data show that males had higher emotional arousal/valence than females for the dark or neutral colour tones (Fig. 2A,B); nevertheless, for the light colour tones, females had higher emotional valence than males (Fig. 2B). The second hypothesis (H2), different interior colour tones evoke higher event-related dynamics in the EEG spectrum with respect to sex differences in emotion, was partially verified by these behavioural results.

### ERSP results

**Figure 3:**
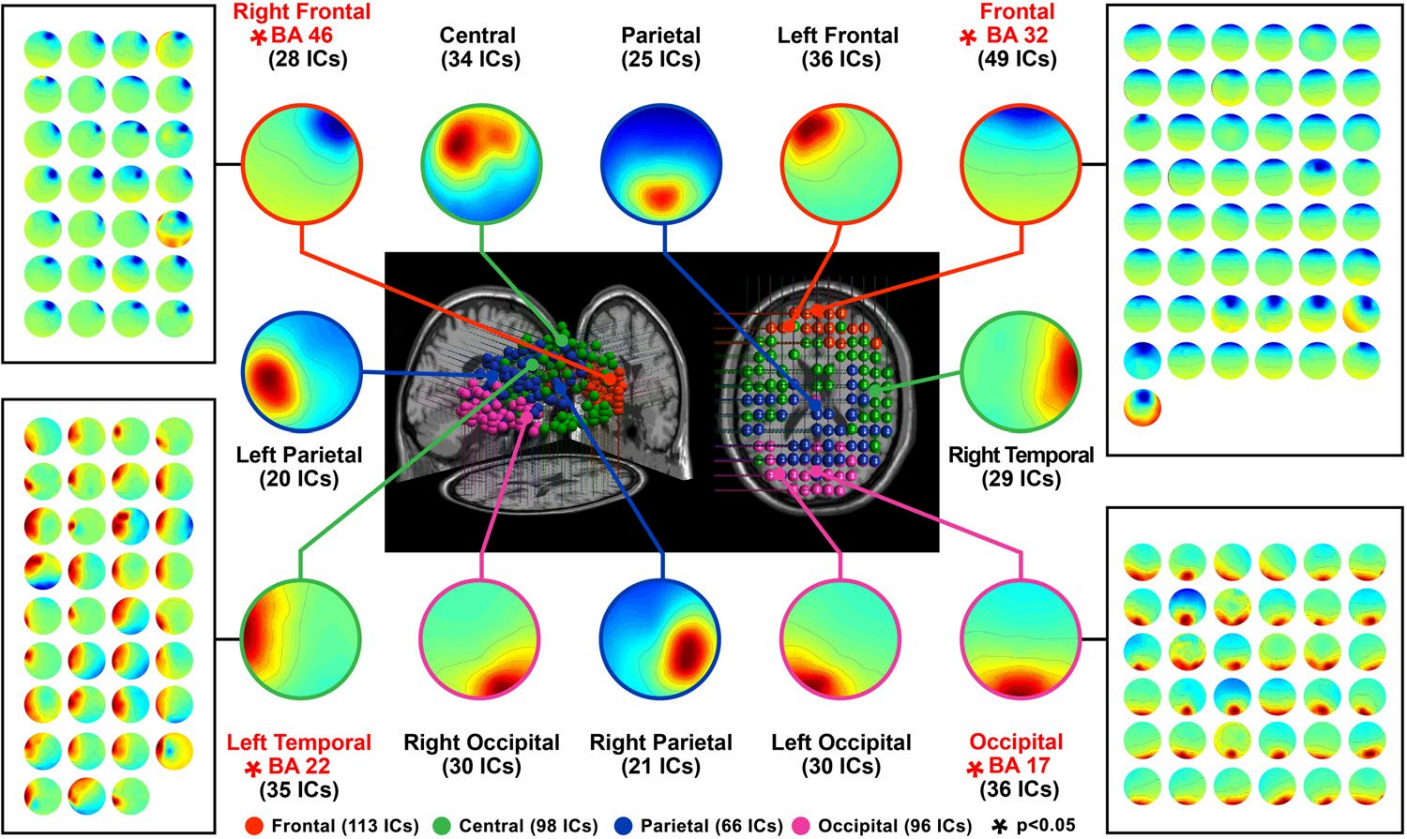
ICA was performed on the EEG signals of the 30 participants (15 males and 15 females) to observe the locations, dipole locations, and scalp map of 12 brain areas. The results showed that significant power changes were concentrated in 4 areas, namely the occipital area (Brodmann Area 17), left temporal area (Brodmann Area 22), right frontal area (Brodmann Area 46), and frontal area (Brodmann Area 32), which showed significant differences in activation power. Significant differences in ERSP were obtained by integrating the differences in the independent variables generated in the various brain regions, which were used to calculate the ERSP images. All images with significant differences in this region were then superimposed. Thus, a deeper colour indicated a richer significant result.

ICA was performed on the EEG signals to analyse the brain areas, dipole locations, and scalp map, as shown in Fig. 3. The event-related spectral perturbation (ERSP) reveals sex differences according to the independent variables used to observe the responsive changes in the event-related brain dynamics^61^.

### Theta, alpha, beta, and gamma activities toward automobile modelling in the occipital, temporal, and frontal areas

**Figure 4:**
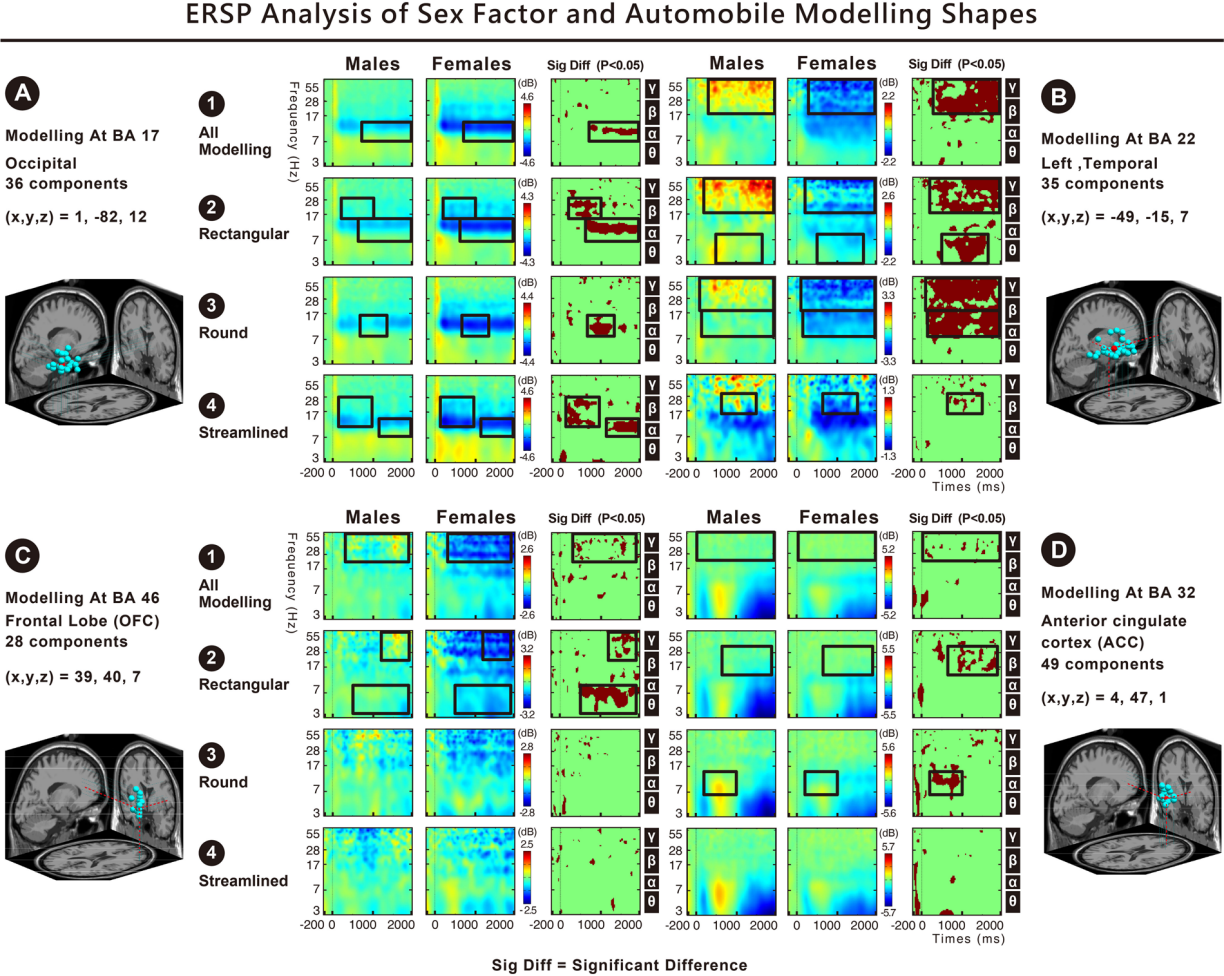
(**A**–**D**) Show the ERSP results of two sexes and automobile modelling. The results indicate that in the occipital area (BA17), left temporal area (BA22), frontal lobe (BA46), and ACC (BA32), the spectral powers of theta (4–7 Hz), alpha (8–13 Hz), beta (14–30 Hz), and gamma (30–50 Hz) showed significant differences (p < 0.05).

We found significant differences in the following brain areas when different sexes (male and female) viewed automobile modelling: Fig. 4A indicates that in the occipital area or Brodmann Area (BA) 17, the alpha-band activation toward all types of modelling, the alpha- and beta-band activation toward all rectangular cars, the alpha-band activation toward round cars, and the alpha- and beta-band activation toward streamlined cars were higher in males than in females. Fig. 4B indicates that in the left temporal area (BA 22), the beta- and gamma-band activation toward all types of modelling; the theta-, beta-, and gamma-band activations toward rectangular cars; and the alpha-, beta-, and gamma-band activations toward round cars were higher in males than in females. Fig. 4C indicates that in the frontal lobe (BA46), the gamma-band activation toward all types of modelling and the theta-band activation toward rectangular cars were higher in males than in females. Fig. 4D indicates that in the (BA 32), the gamma-band activation of females toward all types of modelling was higher than that of males; the beta-band activation of females toward rectangular cars was higher than that of males; and the alpha-band activation of males toward round cars was higher than that of females.

**Figure 5:**
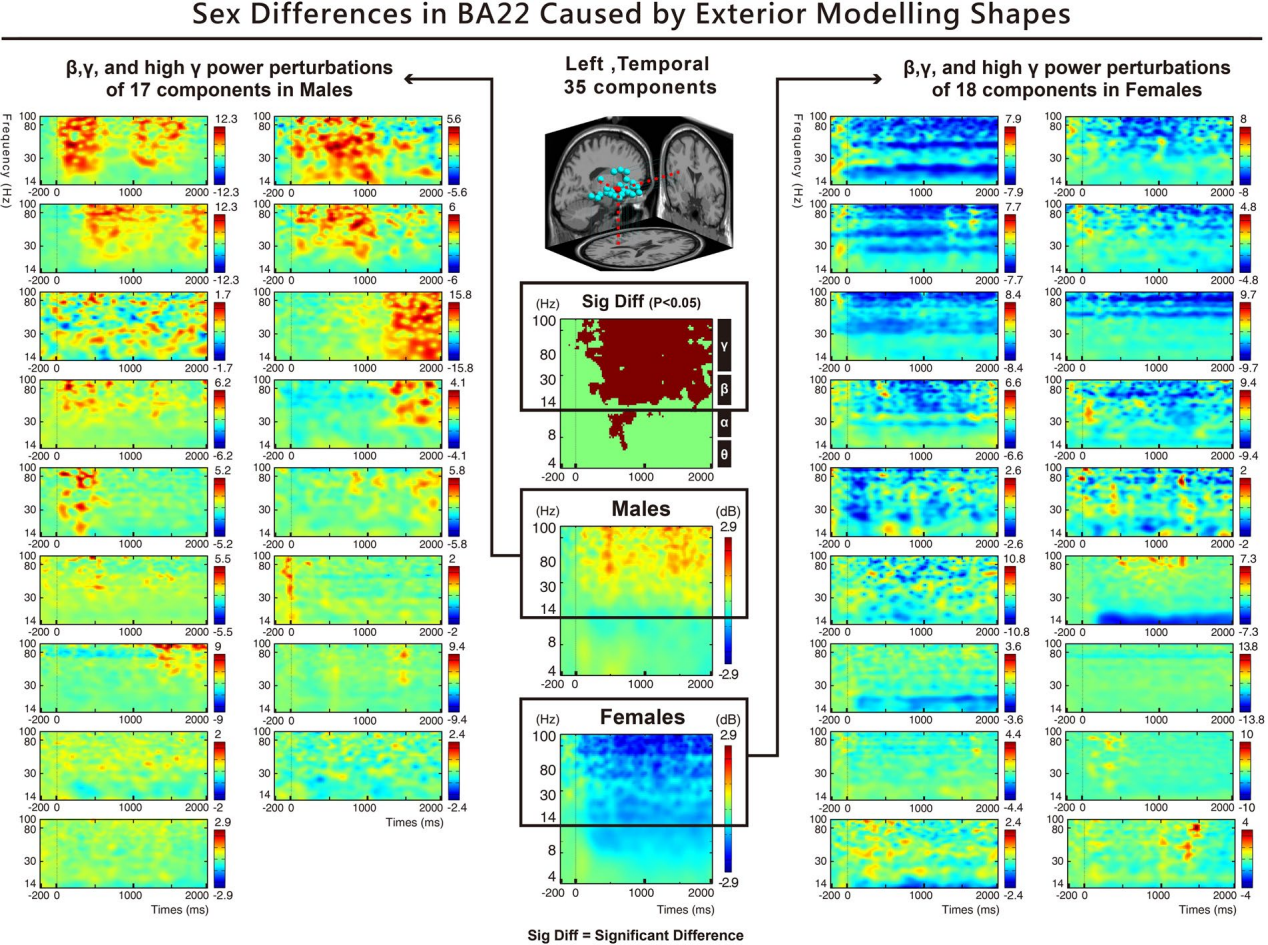
Shows the ERPS results of two sexes and automobile modelling. Significant differences can be observed in the left temporal area (BA22) (FDR-corrected p < 0.05). For all types of modelling, the spectral power of males at 300–2000 ms for 14–100 Hz (beta, gamma, and high gamma bands) was higher than that of females (p < 0.05). Further examination of the 35 components between males and females in the left temporal area (BA22) indicated that 17 components in males showed positive activation in spectral power, while 18 components in females showed significant negative weakening in spectral power.

Furthermore, our observations indicated that, regardless of the car modelling, significant differences could be observed in the high frequency bands of the left temporal area (BA22) between the two sexes. The results showed that the high gamma band at 80*–*100 Hz was significantly different (Fig. 5). Males had a higher power high gamma band response than females for the rectangular and rounded designs of the automobile modelling shapes (Fig. 4).

### Theta, alpha, beta, and gamma activities toward interior colour tones in the occipital, temporal, and frontal areas

**Figure 6:**
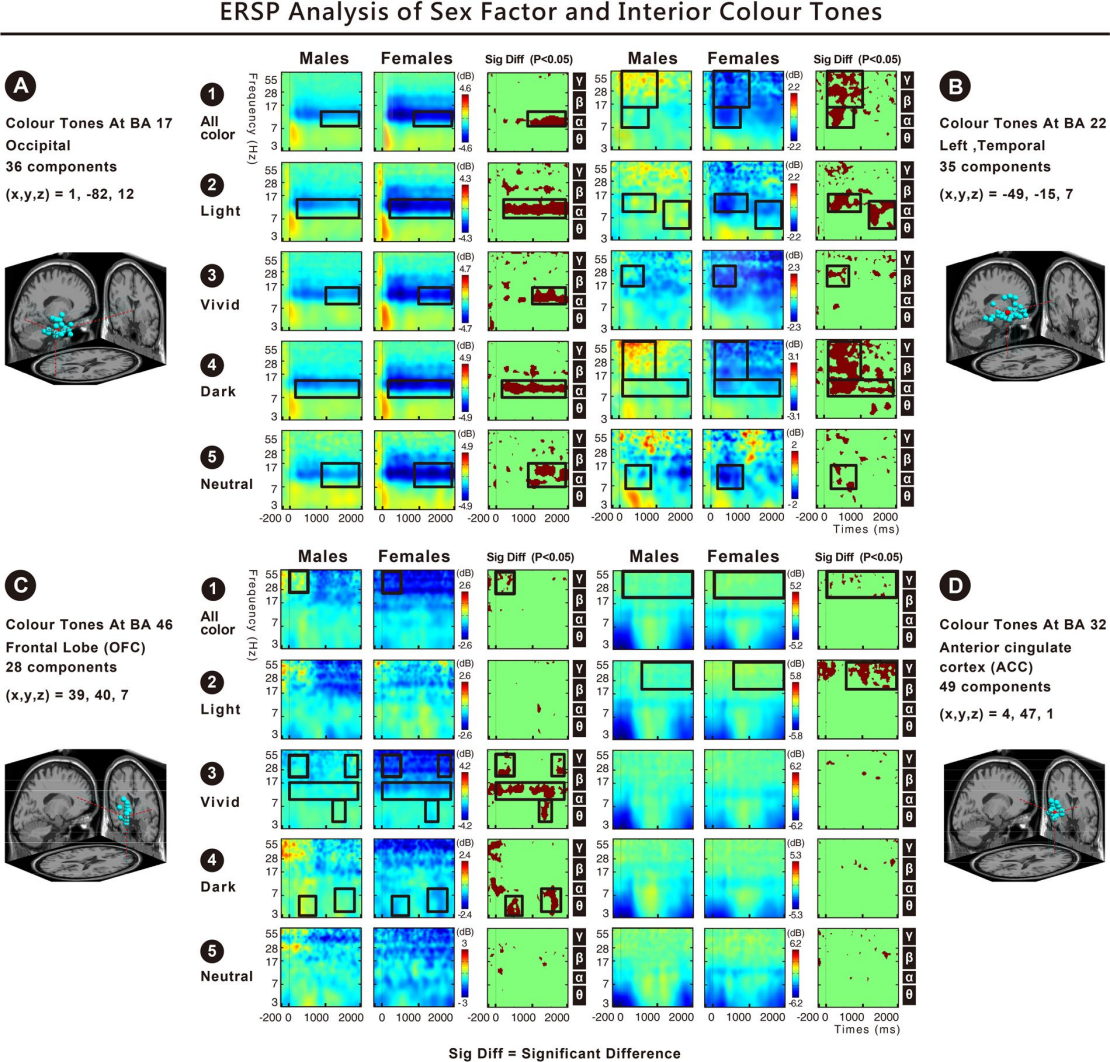
(**A**–**D**) Show the ERSP results of two sexes and interior colour tones. The results indicate that in the occipital area (BA17), left temporal area (BA22), frontal lobe (BA46), and ACC (BA32), the spectral powers of theta (4–7 Hz), alpha (8–13 Hz), beta (14–30 Hz), and gamma (30–50 Hz) showed significant differences (p < 0.05).

The significant differences found in the following brain areas when different sexes (male and female) viewed interior colour tones: Fig. 6A indicates that in the occipital area (BA17), the alpha-band activation toward all colour tones (light, vivid, dark, and neutral) was higher in males than in females. Fig. 6B shows that in the left temporal area (BA22), the alpha-, beta-, and gamma-band activations toward all colour tones (light, vivid, dark, and neutral); the beta-band activation toward vivid colour tones; the alpha-, beta-, and gamma-band activations toward dark colour tones; and the alpha-band activation toward neutral colour tones were higher in males than in females. Fig. 6C indicates that in the frontal lobe (BA46), the gamma-band activation toward all colour tones; the theta-, alpha-, and gamma-band activations toward vivid colour tones; and the theta-band activation toward dark colour tones were higher in males than in females. Fig. 6D indicates that in the ACC (BA32), the gamma-band activation toward all colour tones and the gamma-band activation toward light colour tones were higher in females than in males.

**Figure 7:**
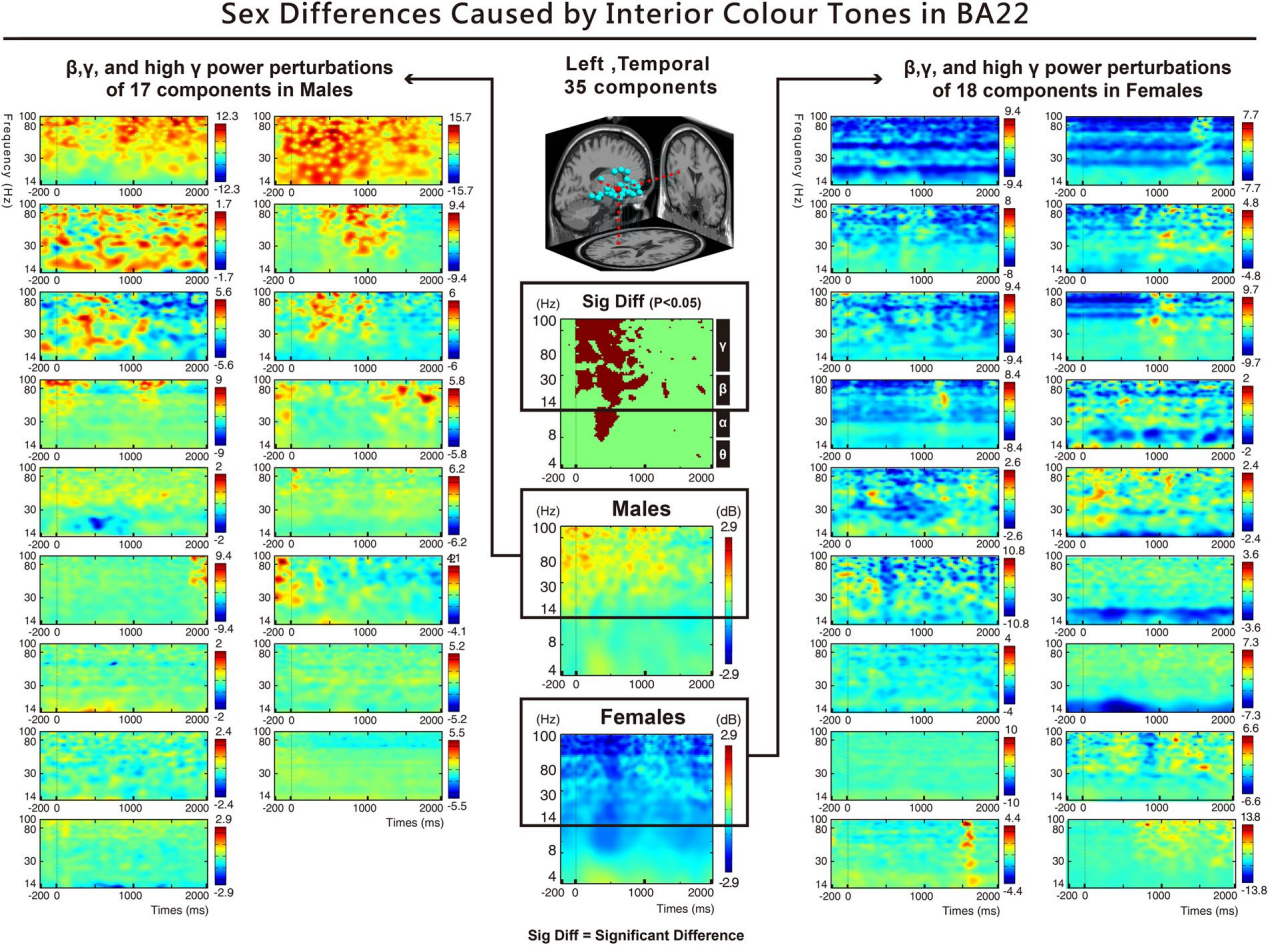
Shows the ERPS results of two sexes and interior colour tones. Significant differences can be found in the left temporal area (BA22) (FDR-corrected p < 0.05). For all colour tones, the spectral power of males at 100–1000 ms for 14–100 Hz (beta, gamma, and high gamma bands) was higher than that of females (p < 0.05). Further examination of the 35 components between males and females in the left temporal area (BA22) indicated that 17 components in males showed positive activation in spectral power, while 18 components in females showed significant negative weakening in spectral power.

Furthermore, our observations indicated that regardless of the interior colour tones, significant differences in the high-frequency bands of the left temporal area (BA22) were found between the two sexes. The results showed that the high gamma-band at 80–100 Hz was significantly different (Fig. 7). Males had a higher power high gamma-band response for the dark colour tone than females (Fig. 6).

**Figure 8:**
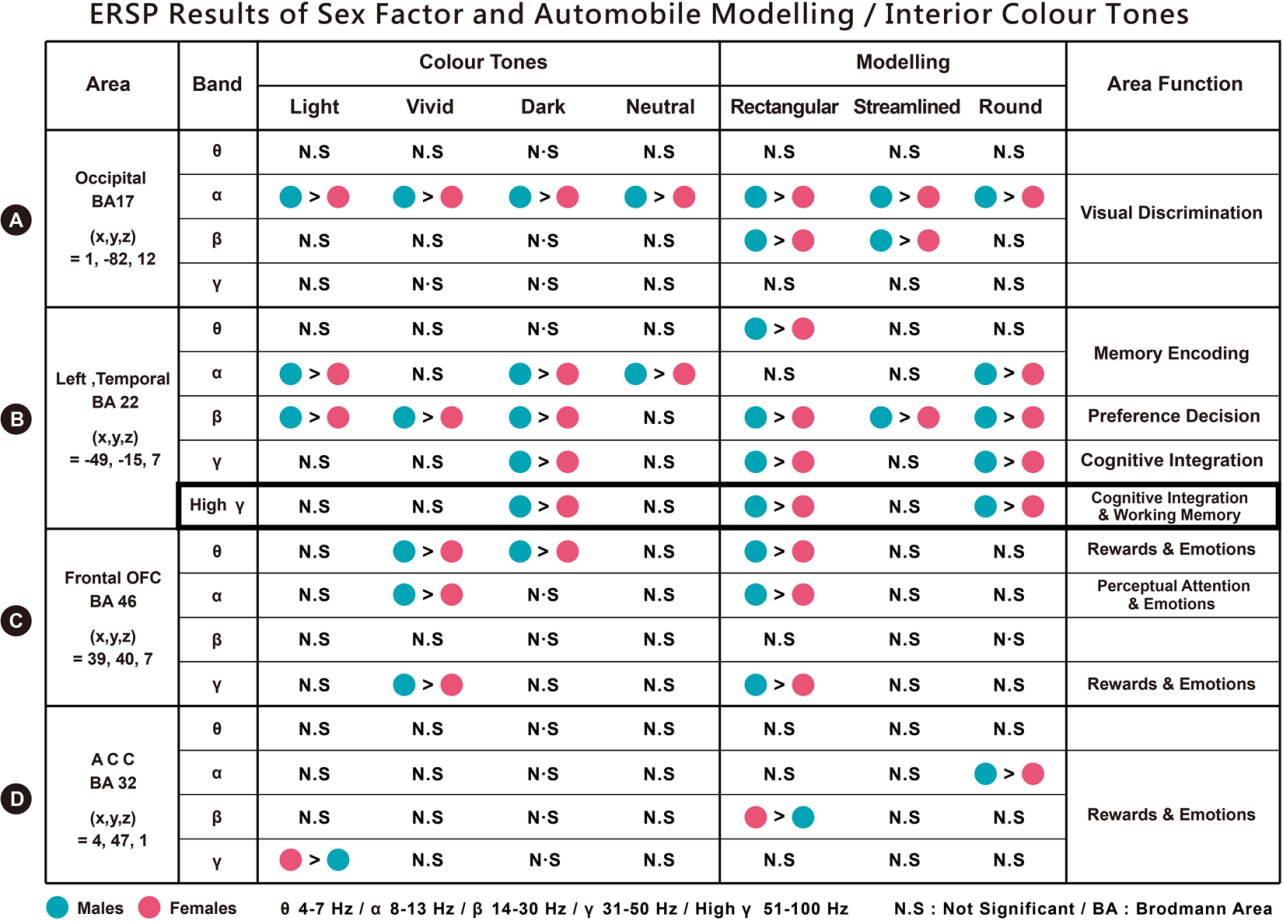
ERSP results indicate significant differences between two sexes toward automobile modelling and interior colour tones in the occipital area (BA17), left temporal area (BA22), orbitofrontal cortex (OFC; BA46), and anterior cingulate cortex (ACC; BA32). (**A**) The significant results in the occipital area (BA17) were as follows: males showed greater alpha band activation toward interior colour tones and greater alpha and beta band activation toward automobile modelling than females. (**B**) The significant results in the left temporal area (BA22) were as follows: males showed greater alpha, beta, gamma, and high gamma band activation toward interior colour tones and greater theta, alpha, beta, gamma, and high gamma band activation toward automobile modelling than females. (**C**) The significant results in the OFC (BA46) were as follows: males showed greater theta, alpha, and gamma band activation toward interior colour tones and greater theta, alpha, and gamma activation toward automobile modelling than females. (**D**) The significant results in the ACC (BA32) were as follows: females showed greater gamma band activation toward interior colour and greater beta band activation toward automobile modelling than males. Males showed greater alpha band activation toward automobile modelling than females.

Sex differences originate from innate physiological division and acquired development. We manipulated two independent variables, namely ‘3D shapes of automobile modelling’ and ‘interior colour tone’ to form 120 stimuli to examine the behavioural data and the human response/EEG event-related dynamics, in terms of individual emotional state, from the two groups of different sex. Most results from the two experimental groups overlapped and were complementary, particularly regarding the car modelling shapes. All results of ERSPs, shown in Figs 4 and 6, were collated into Fig. 8, and the findings were as follows.

(A) The ERSP results indicated significant differences between two sexes toward automobile modelling and interior colour tones in the occipital area (BA17), left temporal area (BA22), OFC (BA46), and ACC (BA32). The comparison of males and females revealed that 3D automobile modelling shapes and interior colour tones evoked higher occipital (BA17), left temporal (BA22), and prefrontal (BA46) activations in males and more anterior cingulate cortex (BA32) activation in females.
(B) The ERSP results along with the behavioural outcomes validate hypothesis H1 and H2 of the study. For the straight or curved lines within the automobile modelling shapes, ranging from rectangular, streamlined, to rounded, males had higher dynamics within the EEG frequency bands for the brain areas, BA17, BA22, and BA46, than females. A smaller number of results indicated that females had higher dynamics in the beta-band of the BA 32 for the rectangular design. The self-reported data showed that the emotional arousal of males was significantly different and higher than that of females for the shapes of automobile modelling. However, each model was not distinct from the others.
(C) For light, vivid, dark, and neutral colour tones, males had a higher power of EEG frequency bands for the brain areas, BA17, BA22, and BA46, than females. Males had higher emotional arousal/valence than females for the dark or neutral colour tones. Fewer results in the present study indicated that females had a higher power gamma-band for the BA32 response to the light colour tone. The results show that females had higher emotional arousal/valence for interior vivid colour tones, but showed reduced cortical activation in all regions.
(D) Our observations of the ERSPs indicated that, regardless of the shapes of automobile modelling and interior colour tones, significant differences in the high-frequency bands of the left temporal area (BA22) were found between the two sexes. The results show that the high gamma band at 80*–*100 Hz was significantly different between males and females. Males had a higher power high gamma band than females for the interior dark colour tone, rectangular, or round design for the automobile modelling shapes.

To facilitate our discussion of the data and validation of the hypotheses of the present study that ‘shapes of automobile modelling’ and ‘interior colour tones’ arouse event-related dynamics in the EEG spectrum with sex differences with respect to the functions of visual processing, rewards, emotions, and information integration mechanism, the following literature are presented.

### Occipital activation and visual processing

Our analysis indicates that males showed greater activation in the occipital area (BA17) than females toward automobile modelling or interior colour tones. The occipital area is the region involved in visual inputs. It mainly processes visual discrimination and integration (e.g., shape, size, and colour). Studies related to colour preferences and colour synaesthesia have shown that visual and colour stimulation activate the occipital area and evoke the activation of theta, alpha, and beta bands^55,56^. By manipulating the shape and pattern of aesthetic packaging design, fMRI studies on aesthetic judgment found that the prefrontal and occipital lobes were evoked^13,62^. This indicates that beautiful visuals can attract the attention of consumers and hence produce higher visual processing. The present study found that males showed greater alpha- and beta-band activation toward automobile modelling in the occipital area (BA17) and greater alpha-band activation toward interior colour tones in the occipital area (BA17) than females. Past studies showed that occipital alpha enhancement indicates the active involvement in the shaping of forthcoming perception, not merely in the simple regulation of visual input^63^. Our experimental stimuli consist of changes in visual features, and males exhibited greater activation than females for different modelling (rectangular/ streamlined/ round) or colour tones (light/ vivid/ dark/ neutral).

### Temporal activation and cognitive comprehension

This study found that males showed greater activation in the left temporal area (BA22) than females toward automobile modelling or interior colour tones. Lengger *et al*. used slow cortical potentials (SCPs) to examine the appreciation of artworks and found that representational information evoked stronger activation in the bilateral temporal lobe^64^. Vartanian and Skov used the *Along the River during the Qingming Festival* painting in an fMRI study on aesthetics and detected the involvement of the temporal lobe, which is involved in semantic processing. The activation of this region indicates the semantic aid provided by aesthetic elements, including styling and clear semantics^51^. The stimuli used in our study and the aforementioned painting share the attribute of clear contour of the objects. In this study, male participants showed greater theta-, alpha-, beta-, and gamma-band activation in the left temporal area (BA22) than females, and gamma-band activation was especially significant. Past studies on episodic memory showed that temporal theta and alpha bands reflect the activation and suppression of episodic information^65,66^. Furthermore, research has shown that gamma-band effects are related to higher cognitive functions, including visual perception, attention, learning, and memory^67^. Experiments on human facial recognition have shown that gamma band (20–90 Hz) activation in the medial temporal lobe (MTL) reflects the maintenance of working memory, and more complex task items will lead to greater activation^68^.

Comprehension involves triggering the visual areas, and beta and gamma responses in the semantic area; meaningful stimuli will evoke a stronger gamma band^54,55^. In addition, studies on the recognition of objects (plants/animals) and surface colour (typical/atypical) have shown that colour is part of the early processing stage in the brain, occurring earlier than the cognitive processing of visual shapes and image semantics. Colour perception will evoke the left occipital and temporal lobes^69^.

### Orbitofrontal activation and emotional rewards

Males showed greater activation of the OFC (BA46) than females toward automobile modelling or interior colour tones. The OFC (BA46) is the brain region for reward processing that is associated with learning and monitoring in terms of the appraisal mechanism of the object qualities. Past EEG studies on reward incentives or fMRI studies on aesthetics showed that both the ACC (BA32) and OFC (BA46) are related to reward processing and emotional monitoring in the brain^70^. This study manipulated the visual features of the experimental stimuli to change the automobile modelling and interior colour tones to enable the participants to experience self-referential cognitive processes, thereby inducing rewards and emotional processing. Males showed greater theta- and alpha-band activation than females in the OFC (BA46) regardless of changes in automobile design (modelling/colour tone). Past studies that used neutral and negative pictures from the IAPS suggested that theta-band activation in the prefrontal cortex is involved in memory and emotional regulation^71^; frontal theta-band enhancement has been regarded as being related to pleasure and emotional arousal from positive emotions^72,73^; the prefrontal alpha band is involved in the attentional and memory inhibition process^71^. Research on preferences for advertising films has found that the prefrontal alpha band can reflect stimuli preference, and higher level of activation implies greater preference^74^. Moreover, a study has shown that happiness, sadness, anger, and other emotional features can induce the increased activation of gamma band, thus indicating that high-frequency activation is related to emotional processing in the brain^75^.

### Anterior cingulate activation and emotional reward

This study found significant differences in the ACC (BA32) activation of males and females toward automobile modelling or interior colour tones. Experiments on aesthetic processing have shown that ACC activation is related to reward and emotional processing^14^. Daily products with beautiful designs and spatial designs with curved forms can evoke pleasant emotions, and activate the participation of the ACC^42,69^. Previous studies have suggested that when viewing visual stimuli with cognitive conflict, the ACC will play the role of detecting irrationality, which is related to the past experiences of the self, while also involving emotions, learning, and memory. Fewer results in the present study show the beta- and gamma-band activations of females to be higher than those of males in the ACC (BA32) toward rectangular cars and interior light colour tones, whereas males had higher alpha-band activation than females in the ACC (BA32) toward round cars. Previous studies using the emotional image task showed that depression-related emotions are reflected in the anterior and posterior alpha-band activation in the brain^76^; aggression and joy are characterized by increased alpha band, while anxiety and sorrow are characterized by decreased alpha band^77^. Furthermore, emotion studies have shown that the reading of positive emotional words evoked the beta band in the ACC, which indicates that the ACC could reflect emotional monitoring, especially its involvement in positive emotional reward. The ACC gamma band provides stimuli for self-awareness^78^. The frontal gamma band indicates cognitive reappraisal of the outcome of the object and modulation of emotional state, which could reflect emotional monitoring^79^. These results could be related to the operations of the brain; that is, the intuitive, quick-thinking System 1 could affect the long-term accumulation of the slow-thinking System 2^80^, thus producing a mutual reciprocal impact in the reward mechanism. The results show that females had higher emotional arousal/valence for vivid colour tone interiors, but they showed reduced cortical activation in all regions. This might be due to less long-term accumulation of automobile experiences in females. These observations may help to explain why many females have difficulties appreciating automotive products.

### Gamma-band activation

Males showed greater gamma-band activation in the left temporal area (BA22) toward automobile modelling and significant late-stage high gamma activation. This indicates that the stimuli for automobile design in this study could evoke high-frequency activity, which could reflect the participation of higher-order sensory and cognitive functions. Furthermore, several studies on gamma band have observed that it is approximately 40 Hz, while a broader gamma band (30–100 Hz) was evoked in this study. Research has shown that broad gamma band activity could reflect more activities in the relevant brain regions^81,82^. Automobile aesthetics led not only to sex differences in cognition but also to perceived emotion. Several previous studies showed that arousal is related to gender. This study found that for both automobile modelling and interior colour tones, males showed greater emotional arousal, which may be related to acquired gender development. In traditional gender development, boys should wear blue clothes and prefer machine or automotive toys, whereas girls should have everything in pink, and like playing house or playing with dolls^6^. Hence, males may be more familiar with automotive products, which would lead to a greater perception and stronger brain activities in response to the aesthetics of different automobile modelling or interior colour tones.

The presence of high-frequency gamma-band activation in the brain has been regarded as preparation to connect and process information received from other regions of the brain. It participates in functions such as sensory processing (visuospatial and auditory), motor control, memory, and attention^81,83^. Moreover, the temporal gamma band is generated in the higher-order cognitive integration of visual processing^84^. Several studies have shown that oscillations at around 40 Hz are intimately related to cognitive and executive functions; frontal gamma band (35–55 Hz) reflects emotional state and monitoring^51,52^, and gamma band (24–35 Hz) in the semantic area is related to the comprehension of visual stimuli^47,48^. Gamma band not only occurs in an extensive range of cognitive and executive processes but also is involved in working memory. Studies on visuospatial recognition (60–80 Hz) have shown that different types of WM require different oscillation frequencies^81^. The alpha-gamma band is related to the maintenance of spatial WM; theta-gamma band oscillations are related to continuous WM information dependency. Automobile purchasing is a highly involved decision that not only involves intuitive and visual emotional arousal but also other reasoning and judgment processes. Aesthetic judgement with high involvement is a subjective process, and includes the ability to utilize knowledge while also involving analysis, planning, decision making, and self-awareness^85^.

Although there is some disagreement that the use of reverse inference is an appropriate exercise for the study of brain activation^86^; cognitive processes can be inferred from functional imaging data if the interpreters carefully take into account the different task settings and responses that have already been studied^86,87^. The predictive power of reverse inference can be determined by the task-setting used^87^. However, there are limitations to the psychophysiological measurements; when combining neuroimaging analysis and self-reported data, which is used in the new field of neurodesign for marketing and aesthetics, the high cost of the research funded by the private sector could mean it is conducted with small sample sizes, which could introduce bias into the results. Nevertheless, the psychophysiological measurement of neurodesign does not rely on consumers to willingly and accurately report their emotions, but focuses instead on physiological responses (brain activations) to specific parts of a design, and so can provide insight into design responses that occur subconsciously. Despite the opinion of scholars, who believe that a small sample size undermines the reliability of neuroscience^88^, the inferential validity of a small sample size in studies is not negligible. If a significant effect can be found with a small sample, it must be caused by a large effect^89,90^. Larger studies may interpret statistically insignificant differences as meaningful^89^. To increase the reproducibility and reliability of the investigation, the experimental methods ought to be detailed, and experimental data should be available to the public^91^.

## Conclusion

The comparison of men and women revealed that 3D automobile modelling shapes and interior colour tones evoked higher occipital, left temporal, and prefrontal activations in men and more anterior cingulate cortex activation in women. Men showed greater theta-, alpha-, beta-, and gamma-band activation in the left temporal area than women, and the gamma-band activation was especially significant and probably indicative of the activation of information on episodic memory. Men might be more familiar with automotive products, which would lead to a greater perception and stronger brain activation in response to the aesthetics of automotive design. They elicited a significantly stronger high gamma band of the left temporal area in response to dark colour tone interiors and to rectangular or round designs of automobile modelling shapes. Men were instructed in the representation of automobile design with clear presentation and semantics and they showed meaningful and comprehensible reactions associated with working memory mechanisms. Comparatively, fewer results showed that women had higher dynamics in the beta-band of the anterior cingulate cortex for rectangular design of automobile modelling shapes, and in the gamma-band for light colour tone interiors, which may relate to their higher self-awareness of the positive emotional reward. The results show that women had a higher emotional arousal/valence for vivid colour tone interiors but reduced cortical activation in all regions. This might be due to less long-term accumulation of automobile experiences in women. These observations help explain why many women have difficulty appreciating automotive products.

## Methods

### Participants and Stimuli

Thirty participants with university degrees or higher were recruited online (15 males and 15 females; aged between 22 and 27 years; mean age, 24.3 years). This study involved a survey of attitudes during the recruitment of participants, ahead of the EEG experiment. All the selected participants had neutral attitudes to all brands of car models in the auto industry. This was assessed using the Likert 5-point scale measurement without presenting any photos of car models or auto brands: from 1 to 5, (1) strongly agree, (2) agree, (3) neutral, (4) disagree, (5) strongly disagree. The stimuli of car modelling in the experiment were presented as idealistic interior colour tones and line drawings without branding to avoid brand association.

Estimation of the achieved power for the recruited sample was advisable^92^, particularly when the sample size was not large^88^. A statistical power post hoc analysis was conducted by calculating the Cohen *d*^93^ using the G*Power 3.1 software to determine the level of power that the main outcome (i.e., emotional arousal/ valence) of the present study achieved. Assuming a medium effect size of Cohen’s d, 0.5, or a medium effect size of Cohen’s f, 0.25^94,95^, for an alpha level of 0.05, a sample size of 24 participants was required to achieve an adequate power of 0.80. Given the sample size (N = 15) for two different sex groups, four for the experimental treatments (i.e., interior colour tones or car modelling) of the present analyses; the achieved power was 0.575 for between-within repeated-measures analysis of variance (ANOVA). The relatively power low achieved is discussed as a limitation of the present study.

The participants had corrected visual acuity of ≥0.8, no colour blindness, no visual impairments, and no history of neurological or psychiatric diseases. The participants did not have drug or alcohol addiction, and were asked to avoid the use of stimulants (e.g., alcohol and caffeine) that might affect their EEG results 48 hours before the experiment. This study was conducted according to the Declaration of Helsinki (World Medical Association, 1964; 2013). Written informed consent was obtained from the participants prior to the experiment, and the study was approved by the Institutional Review Board for Human Trials. The study was approved by the human trial institutional review board of Cathay General Hospital. All methods were performed in accordance with the approved guidelines. Informed consent was obtained from all participants prior to the experiments.

Automobile brands from 2011 to 2014 (Audi, BMW, Citroën, Chevrolet, Ford, Fiat, Daihatsu, Honda, Hyundai, Kia, Mazda, MINI, Mitsubishi, Nissan, Opel, Perodua, Peugeot, Renault, SEAT, Skoda, Suzuki, Toyota, and Volkswagen) were collected, and 55 left-driving models with carbon emissions below 1600 cc were selected as reference for designing the experimental stimuli. The independent variables were automobile modelling and interior colour tones. The 3D shapes of the automobile modelling were presented as line drawings: (a) rectangular (few curves), represented by the MINI ONE; (b) streamlined (moderately curved), represented by the BMW 1 series; and (c) round (very curved), represented by the Volkswagen Beetle (Fig. 1B). The brand logos were removed from the stimuli to avoid the interference of the brands through subjective association. The colour tones were designed as follows: light tone, vivid tone, and dark tone. Each colour tone included 12 hues (R, Ro, yO, Y, yG, G, BG, gB, B, V, P, and RP) of the PCCS, which formed the experimental group. The neutral colour tones were of 4 hues (itGy, mGy, dkGy, and Bk), which formed the control group (Fig. 1C). The experimental group of stimuli consisted of nine conditions as follows: (rectangular and light tone), (rectangular and vivid tone), (rectangular and dark tone), (round and light tone), (round and vivid tone), (round and dark tone), (streamlined and light tone), (streamlined and vivid tone), and (streamlined and dark tone). Stimuli for the experimental groups were created for each condition on the basis of the 12 hues of the PCCS to give 108 stimuli (12 hues × 9 conditions). The control group consisted of 3 conditions as follows: (rectangular and neutral colour), (round and neutral colour), and (streamlined and neutral colour). Each neutral colour tone was created based on the four neutral colour hues of the PCCS to give 12 stimuli. The 10 conditions of stimuli were the nine experimental groups and one control group. Each condition had 12 stimuli. A total of 120 stimuli were repeated three times, which made a total of 360 trials. Thirty-six trials were conducted for each condition (120 stimuli × 3 times = 360 trials/10 conditions = 36 trials per condition). All the colour tones of the stimuli in the experiment were applied to the dashboard, seat, and door trim panel, and the other interior features remained as black.

### Procedure

This study was conducted in the National Taiwan University of Science and Technology Design Perceptual Awareness Laboratory (D:PAL Lab). External interferences (e.g., noise, temperature, and light) were strictly controlled. During the experimental process, the participants provided their answers alone in the laboratory. The screen was placed on a 74-cm-high table, and the centre of the screen was placed within 10°–20° of the participants’ line of sight, at a distance of 60–70 cm from the participants. The experimental equipment used was the US Neuroscan EEG recording and analysis system (Scan 4.3.3 & STIM2), which included an electrode cap (Quik-Cap) and amplifier (SynAmps2, Compumedics Ltd., VIC, Australia). Sixty-four-channel EEG signals were recorded in accordance with the international 10–10 system of electrode placement. The experimenter observed the participants’ condition and EEG recoding status from a screen outside the laboratory. After the participants finished reading the instructions provided, the formal experimental procedure began. A ‘+’ fixation point was presented for 1000 ms at the start of the procedure, which prepared participants for the EEG experiment. This was followed by the presentation of car modelling stimuli for 2000 ms (S1) and then by interior colour tone stimuli for 2000 ms (S2). The 120 stimuli were repeated 3 times and randomly presented on the computer screen (1024 × 768 pixels) to examine the participants’ EEG responses. After viewing the experimental stimuli, the participants were presented with the following behavioural questionnaire: ‘What do you think is the level of emotional valence produced by the aesthetics of this car?’ and ‘What do you think is the level of emotional arousal produced by the aesthetics of this car?’ The response options were designed by combining the Likert 5-point scale and the Self-Assessment Manikin (SAM), where ‘1’ is the lowest level and ‘5’ is the highest level. A total of 360 stimuli were presented. A rest period was given after every 120 stimuli, and the participants decided the length of the rest period before continuing the experiment. The total length of the experiment was about 45 minutes.

## Data analyses

### Behavioural data analysis

The Latin square experimental design^96^ was used in this study, which separates the effects of a single factor from the experimental stimuli generated from all combinations of the independent variables, thus allowing us to observe the effects of this independent variable on the dependent variables. The *n*x*n* Latin square experimental design included *n* types of treatments in two factors (A1 to An, and B1 to Bn). Behavioural data were analysed using two-way analysis of variance (ANOVA) according to the independent variables, whereby ‘modelling’ and ‘colour tone’ were manipulated to observe their effects on the emotional dimensions of different sexes. The Bonferroni method was used for post hoc testing. Numerous types of EEG studies have used the Latin square experimental design, which has been applied in auditory, visual discrimination tasks, and emotion studies^97–99^.

### Independent component analysis and clustering

The MATLAB (The Mathworks, Inc.) open-sourced toolbox EEGLAB (Swartz Center for Computational Neuroscience, La Jolla, CA; http://www.sccn.ucsd.edu/eeglab) was used to analyse the 64-channel continuous EEG data. Raw EEG data were filtered using a 1-Hz high-pass filter and 100-Hz low-pass filter. Line noise was removed (60 Hz and its harmonic). After filtering, the signal sampling rate was reduced to 250 Hz to save on storage space and number of operations.

Independent component analysis (ICA) in EEGLAB was used^99^ (Delorme and Makeig, 2004) to decompose the recorded EEG signals into independent components (ICs) generated by different brain regions^100^. ICA can decompose *N* EEG signals into *N* ICs, and the ICs of the 30 participants were clustered into common brain regions as follows: left frontal area, frontal midline area, right frontal area, left temporal area, central area, right temporal area, left parietal area, parietal area, right parietal area, left occipital area, occipital area, and right occipital area). The Talairach *xyz* coordinates^101,102^ of the area centroids were matched to the Brodmann regions to understand the actual brain functions of these regions.

### Event-related spectral perturbation

After ICA, the ICs were converted to event-related spectral perturbation (ERSP) images. The signals recorded from the statistically significant electrodes were decomposed by a sinusoidal wavelet transformation^103–105^. One advantage of wavelet analysis over short-time Fourier transform is that the window size can be varied according to the frequency being analysed^14,71^. The default settings of ‘wavelet cycles’ in EEGLAB were [3 0.8], in which the wavelet analysis applied a 3-cycle wavelet at the lowest frequency and a 0.8-cycle wavelet in the window length at the highest frequencies to EEG epochs. Overlapping Hanning tapered windows were applied. The analysis procedure estimated 100 log-spaced frequencies from 3.0 to 125.0 Hz. The wavelet analysis provided a more flexible approach to optimize the temporal resolution of different frequencies of the EEG signals. Owing to the 100-Hz low-pass filtering, we obtained a 3- to 100-Hz frequency in the responses to the wavelet transformation. The perturbations from baseline were colour coded^99^. ERSP intensity is directly proportional to colour intensity; a deeper colour indicates a stronger oscillatory response. After the stimuli were presented, positive oscillations were presented as red in ERSP; conversely, negative oscillations were presented as blue. In the ERSP image, green indicates no significant difference in EEG oscillatory activity compared with that at baseline. The stimuli of interior colour tone and automobile modelling were viewed for 0–2000 ms, the data of all trials were then averaged to obtain the ERSP image to observe the theta (4–7 Hz), alpha (8–13 Hz), beta (14–30 Hz), low gamma (31–50 Hz), and high gamma (51–100 Hz) bands, and to match the neurophysiological implications of each brain region. The length of time was determined from the pilot test of all participants, and was sufficient for them to see and interact with the stimuli during its presentation^106^. The ERSP presented various aspects of brain dynamics associated with the events, and these are not included in the average event-related potential (ERP) of the same response epochs^61^. Finally, the data were grouped according to the independent variables to compare the ERSP and determine any statistically significant differences (*p* < 0.05); between-group differences were determined using *T* tests. The *p* values were adjusted using the false discovery rate (FDR)-controlling multiple testing procedure (the fdr.m routine from the EEGLAB toolbox)^107^.
